# Harnessing Motile Amoeboid Cells as Trucks for Microtransport and ‐Assembly

**DOI:** 10.1002/advs.201801242

**Published:** 2018-11-28

**Authors:** Oliver Nagel, Manuel Frey, Matthias Gerhardt, Carsten Beta

**Affiliations:** ^1^ Institute of Physics and Astronomy University of Potsdam Karl‐Liebknecht‐Str. 24/25 14476 Potsdam Germany

**Keywords:** biohybrid microsystems, chemotaxis, *Dictyostelium discoideum*, microtransport and ‐assembly

## Abstract

Cell‐driven microtransport is one of the most prominent applications in the emerging field of biohybrid systems. While bacterial cells have been successfully employed to drive the swimming motion of micrometer‐sized cargo particles, the transport capacities of motile adherent cells remain largely unexplored. Here, it is demonstrated that motile amoeboid cells can act as efficient and versatile trucks to transport microcargo. When incubated together with microparticles, cells of the social amoeba *Dictyostelium discoideum* readily pick up and move the cargo particles. Relying on the unspecific adhesive properties of the amoeba, a wide range of different cargo materials can be used. The cell‐driven transport can be directionally guided based on the chemotactic responses of amoeba to chemoattractant gradients. On the one hand, the cargo can be assembled into clusters in a self‐organized fashion, relying on the developmentally induced chemotactic aggregation of cells. On the other hand, chemoattractant gradients can be externally imposed to guide the cellular microtrucks to a desired location. Finally, larger cargo particles of different shapes that exceed the size of a single cell by more than an order of magnitude, can also be transported by the collective effort of large numbers of motile cells.

The directed transport of micrometer‐sized objects in complex confined environments is a challenging task in many areas of technology, life science, and medicine. Examples include the construction of microfluidic devices and microelectromechanical systems but also medical applications such as the deposition of drug releasing capsules in specific places in the human body.^1^ Complex laborious approaches are available to perform such tasks, typically relying on advanced microfabrication techniques, on external force fields such as optical or magnetic tweezers, or on microsurgery.^2^ Alternatively, we may also envision small microrobots that are programmed to fulfill such transport tasks in an autonomous way. However, despite considerable progress, the performance of artificial micromachines is still severely limited.^1^, ^3^

In a recently emerging approach to enhance the performance of microsystems, artificial components are combined with biological cells to a functional device. Such biohybrid systems rely on the intrinsic actuation and sensing functionalities of biological cells that still outperform most artificial, synthetically constructed micromachines. One of the most fundamental functions that motile cells can contribute to a biohybrid device is their ability to move micrometer‐sized objects in a directed fashion. The femtowatt power generated by such devices is too low for operating macroscopic machines. However, the ability of microorganisms to actuate sub‐millimeter objects provides a promising basis for novel micromanipulation and positioning techniques.^3^, ^4^ To date, this has been demonstrated in several contexts. For example, collectively swimming bacteria may actuate small mechanical devices.^5^ Swimmers that get trapped by an entropic effect inside the funnel‐shaped structures of an asymmetric gear wheel effectively transfer momentum to the gear by collisions. This leads to a nonzero net momentum of the system and thus results in turning of the wheel.^6^ Moreover, various biohybrid microswimmers have been created by attaching microcargo to swimming algae^7^ and sperm cells,^8^ as well as to various bacterial swimmers, such as *Escherichia coli* 9 or *Vibrio alginolyticus*.^10^ Future applications of such “bacteriobots” can be expected in the field of drug delivery. For example, it was recently shown in a tumor mouse model that microbeads driven by *Salmonella typhimurium* have tumor targeting abilities and accumulated near the tumor site.^11^ For a review of biohybrid microtransporters see Ref. 3.

Despite these successful examples, research on cell‐driven microtransport is still at an early stage. In particular, previous work on cellular microtransporters was mostly focused on swimming motility. To the best of our knowledge, microtransport by adherent eukaryotic cells that crawl on solid substrates or through narrow channels has not been explored. A motile eukaryotic cell can be seen as a functional unit, built up from a large number of efficient nanodevices like polymerization ratchets and cytoskeletal motors. The cell orchestrates this complex machinery to move in a coordinated and directed fashion. Given the prominent role of crawling cells in development, functions, and diseases of the human body, their potential as microtransporters is a highly relevant avenue to explore. In light of the recent advances in understanding motility and chemotaxis of adherent cells, this is a timely undertaking and a logical next step. Compared to swimmers, adherent cells are less prone to flow effects. They offer a variety of options for directional guidance by chemotactic cues,^12^ their properties can be tuned with the help of genetic tools, and they show diverse effects of self‐organization and collective behavior that can be exploited to fulfill complex transport tasks.

Here, it is our aim to explore the potential of motile amoeboid cells to act as microtrucks that move their microcargo in an independent, self‐organized fashion. In particular, we will show that (a) motile amoeba can be loaded with microcargo of different size and shape, (b) cell‐driven transport of the microcargo can be controlled and guided by chemotactic signals, and (c) several cells can move cargo particles collectively if the cargo dimensions exceed the transport capacities of individual cells. We present results that yield a clear proof‐of‐principle for this novel paradigm of cell‐driven microtransport. Our experiments were performed with cells of the social amoeba *Dictyostelium discoideum*, a well‐established eukaryotic model organism for actin‐driven motility and chemotaxis. *Dictyostelium* shows many homologies to mammalian cells, such as neutrophils or cancer cells.^13^ In particular, its locomotion and chemotactic performance has been quantified at different levels, including the underlying chemotactic signaling cascade^14^, ^15^ as well as the statistics of its trajectories.^16^, ^17^

*Dictyostelium* cells bind unspecifically to a wide variety of substrates, such as naked glass or polystyrene surfaces. Although no integrins have been found in the *Dictyostelium* genome, several other proteins were identified that affect *Dictyostelium* adhesion, such as the actin anchoring protein talin,^18^ the nine‐transmembrane proteins Phg1^19^ and SadA,^20^ the disintegrin domain protein AmpA,^21^ and the type I transmembrane protein SibA.^22^ Nevertheless, adhesion is largely unspecific and does not require any specific surface coatings. In particular, *Dictyostelium* cells move on hydrophobic as well as hydrophilic surfaces,^23^ which has led to the hypothesis that adhesion is primarily mediated by Van der Waals interactions between cell surface glycoproteins and the substrate surface.^24^ *Dictyostelium* is thus a well‐suited candidate to carry objects of different materials without requiring elaborate surface treatment. In the course of our experiments, polystyrene particles, agar particles coated with wheat germ agglutinin, and custom‐made objects composed of SU‐8 2025 photo resist were used without any noticeable difference in the adhesive properties.

In a first series of experiments, we incubated vegetative *Dictyostelium* cells together with polystyrene microbeads of 15 µm in diameter. Even in the absence of stimuli, *Dictyostelium* cells are intrinsically motile and perform an isotropic random walk with an average speed of about 4 µm min^−1^.^25^ The microparticles remain stationary as long as they are not in contact with a cell, see Movie S1 in the Supporting Information. As a control, we have also imaged microparticles of 10 µm in diameter in the absence of cells to demonstrate that movements by colloidal forces is negligible, see Movie S2 in the Supporting Information. In the course of their random motion, cells eventually come into contact with one of the neighboring microparticles. Due to the unspecific adhesive interactions, the particle may stay attached to the cell surface and may be dragged along as the cell moves on, see Movies S1 in the Supporting Information for examples. While details of the cell‐particle contact are difficult to image, we quantified the success rate of collisions by analyzing a total number of 44 events, where the outline of the cell overlapped with the projected circumference of the cargo particle. In 24 cases (55%), the particle was successfully attached and carried away by the cell, while in the remaining cases the particle stayed in its initial position and the cell moved away without cargo.

In **Figure** **1**A,B (Movie S3, Supporting Information), the detailed recording of a successful particle uptake is shown. The blue path displays the center of mass trajectory of a randomly moving *Dictyostelium* cell before it comes into contact with a microparticle. After the collision event, the particle is moving along with the cell, its trajectory is shown in orange in Figure 1, where panels (A) and (B) are the first and last frames of the recording, respectively. Note that the trajectory of the cell center is only shown until the collision event. Cells may also lose their cargo particles. However, due to the finite time of our recordings, the complete trajectories including both uptake and loss events were captured only for part of the transported cargo (22%). In many cases, cells were already carrying their cargo at the beginning of the recording, or they were still carrying it when the recording ended. Based on the method proposed by Vardi,^26^ we estimated a mean cargo attachment time of 224 min from a censored data set of 49 cargo trajectories. The shortest trajectory was 9.5 min long, while the longest exceeded the total measurement time of 360 min.

**Figure 1:**
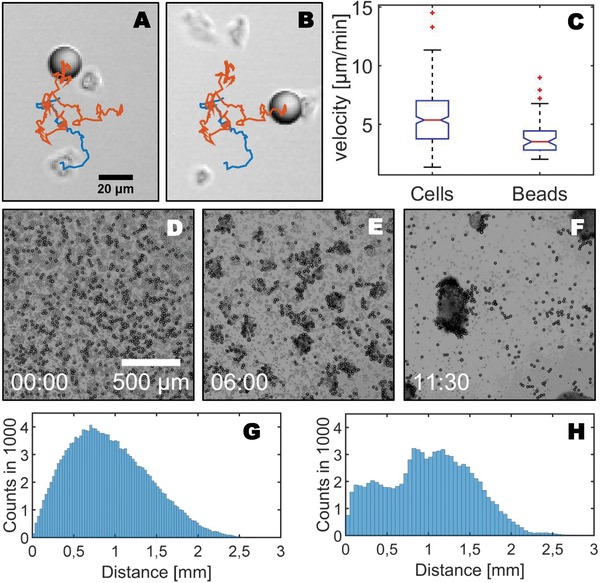
Transport and assembly of microparticles by spontaneously moving and aggregating cells. A,B) Trajectory of a cell (blue) before it encounters a polystyrene microbead (20 µm in diameter) and trajectory of the microbead (orange) carried along by the cell after the encounter. Panels (A) and (B) display the first and last frames of the recording, respectively. C) Average speed of 313 individual *Dictyostelium* cells that do not carry a microbead compared to the average speed of 62 microbeads that are carried by single *Dictyostelium* cells. D–F) Assembly of microbeads driven by the spontaneous aggregation of developing *Dictyostelium* cells. Histograms of the distances between the microbeads G) at the beginning and H) at the end of the recording are shown. They correspond to the snapshots shown in panels (D) and (F).

We have tracked the motion of 62 microbeads that were carried by a single *Dictyostelium* cell and compared their averaged speed to the speed of 313 cells that did not carry any microcargo and where recorded during the same experiments. We found an average speed value of ≈5.5 ± 2.3 µm min^−1^ for cells without beads and one of ≈4 ± 1.5 µm min^−1^ for beads carried by a single cell, see Figure 1C. This indicates that the overall motility of an amoeboid cell is only moderately affected by a cargo particle measuring 20 µm in diameter. We also note that for a particle diameter of 20 µm we did not observe any cells that moved more than one particle at a time. This is most likely due to steric reasons. For smaller cargo dimensions cells can move several particles at once. For particles of 10 µm in diameter single cells were observed to carry one or two particles at a time, and in our experiments with particles of 4.5 µm in diameter, cells were carrying 6 ± 2 cargo particles on average, see Figure 2D,E and Movies S7 and S8 in the Supporting Information.

*D. discoideum* is famous for its multicellular life cycle.^27^ When deprived of food, populations of *Dictyostelium* cells spontaneously aggregate to form large multicellular assemblies that eventually transform into a differentiated fruiting body. The initial aggregation relies on cAMP chemotaxis and progresses via the formation of cell clusters and streams that line up and jointly flow toward a common aggregation center. Can we exploit this collective phenomenon to drive the aggregation of microparticles in a self‐organized manner? We have incubated *Dictyostelium* cells together with polystyrene beads in phosphate buffer to initiate starvation‐induced development. Similar to our recordings in growth medium, we observed that cells picked up microparticles and carried them along while randomly moving across the glass surface. During starvation, cells started to assemble into small multicellular clusters that eventually merged into larger assemblies. During this self‐organized aggregation process, most of the microbeads remained attached to the cells and were carried along towards the aggregation centers. In Figure 1D–F, an example of this process is shown for polystyrene beads measuring 20 µm in diameter. The aggregating cells assembled the initially uniformly distributed beads into localized clusters, see also Movie S4 in the Supporting Information. We thus conclude from our observations that the early aggregation phase of the developmental cycle is not altered by the presence of the microbeads. *Dictyostelium* cells aggregate normally in the presence of micro‐objects, so that their self‐organized assembly into clusters can be successfully exploited to accumulate such micro‐objects in an autonomous fashion. We quantified this aggregation process by measuring the distance from each particle to all other particles in the field of view. A distribution of these distances can be seen in Figure 1G for the beginning of the recording and in Figure 1H for the final pattern of microbeads after 11.5 h. The initial distribution with a pronounced peak at 0.65 mm (0.5 × *L*, with *L* = 1.3 mm the side length of the field of view) transforms into a bimodal distribution, where the first peak represents the distances between particles inside the aggregates, while the second peak reflects the distances between different aggregates.

The aggregation of developing *Dictyostelium* cells relies on cAMP‐mediated chemotactic signaling. During the first hours of starvation, cells express cAMP receptors and reliably migrate towards increasing concentrations when exposed to a cAMP gradient. They sense gradients over several orders of magnitude, ranging from shallow signals close to the noise level to steep gradients and high offset concentrations.^28^ Can we exploit the chemotactic properties of these motile amoeboid cells to transport microcargo in a directed fashion to a desired location?

In a first set of chemotaxis experiments, we used the commercially available ibidi “µ‐Slide Chemotaxis” gradient chamber to expose starvation‐developed *Dictyostelium* cells to a linear cAMP gradient established by diffusion between two reservoirs, see the Supporting Information for a detailed layout of the chemotaxis chamber. In this case, cells were incubated together with agar particles that were coated with wheat germ agglutinin (WGA) and ranged in size up to 50 µm in diameter. While moving up the gradient, cells may randomly collide with the agar particles. When coming into contact with a cell, the agar particles remained attached to the cell membrane and were dragged along by the moving cell in gradient direction. An example of this directed transport is shown in **Figure** **2**A, where the trajectory of a particle with a diameter of ≈40 µm is displayed, see also corresponding Movie S5 in the Supporting Information. Note that single cells were able to transport particles which exceeded their own size by a factor of five, see also the inset in Figure 2A. We also observed that in this case binding of the particles to the cells was not irreversible. In some cases, cells lost their cargo, which was then picked up by succeeding cells. Thus, the chemotactic flux of cells sets an upper limit to the overall flux of particles in gradient direction, which will in general be lower.

**Figure 2:**
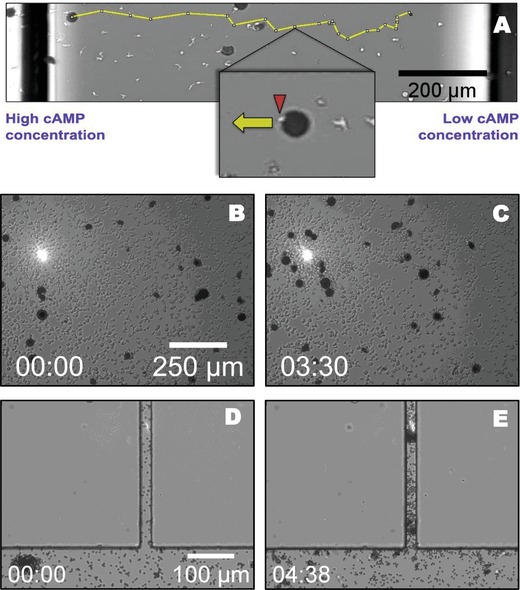
Directionally guided transport of microparticles by chemotactic cells. A) Transport of WGA coated agar particles in a stationary linear gradient of the chemoattractant cAMP. In yellow, the trajectory of a 40 µm particle is shown that is carried by a single *Dictyostelium* cells. B,C) Transport of WGA coated agar particles toward a point source of cAMP created by photouncaging of BCMCM‐caged cAMP in a 366 nm laser spot. D,E) Directional transport of 4.5 µm polystyrene particles into the narrow side channel of a microfluidic chamber, where cAMP is released by photouncaging of BCMCM‐caged cAMP.

Diffusion chambers that produce stationary linear gradients pose several limitations on the type of transport task that can be fulfilled in such a setting. In particular, it is difficult to change the steepness and direction of the chemoattractant gradient in such chambers during the experiment, so that the transport direction remains fixed over time. Also the maximal distance over which objects can be moved is limited by the width of the diffusion chamber and by saturation of the chemoattractant receptors that will eventually become relevant, if cells move in a continuously increasing gradient profile. Moreover, in many settings it will be technically difficult to accommodate the two large reservoirs that are required for diffusive buildup of the chemoattractant gradient.

To overcome some of these limitations, we introduce a chemoattractant point source that can be flexibly repositioned during the experiment. In order to access also closed microenvironments of complex geometry, we propose the use of photoactivatable chemoattractants that can be released by a laser point source.^29^, ^30^ To demonstrate this concept with our amoeboid model organism, we used the commercially available [6,7‐bis(carboxymethoxy)coumarin‐4‐yl]methyl (BCMCM)‐caged form of cAMP that was uncaged in the focused spot of a 366 nm laser light source.^31^ In Figure 2B,C, snapshots from an experiment are shown, where starvation developed *Dictyostelium* cells were incubated together with WGA‐coated agar particles in a glass‐bottom Petri dish under phosphate buffer containing 10 µm of BCMCM‐caged cAMP. When switching on the laser point source in the upper left hand corner, cAMP was locally released by photouncaging and a concentric gradient profile built up in the vicinity of the laser spot. Under the influence of this gradient, the chemotactic cells moved towards the light source and drag the agar particles along, as is evident from the increased particle density in the vicinity of the laser spot in Figure 2C, see also the corresponding Movie S6 in the Supporting Information.

We also demonstrated that this approach can be successfully used to navigate microcargo inside closed microfluidic chambers. We have incubated chemotactic *Dictyostelium* cells together with polystyrene beads of 4.5 µm in diameter in phosphate buffer containing 10 µm of BCMCM‐caged cAMP and inserted the suspension into a microfluidic channel with narrow dead‐end side branches, see Figure 2D. By positioning the focused 366 nm laser spot in one of the side branches, cAMP was photochemically released to establish a concentration gradient along the side branch and into the main channel by diffusion. *Dictyostelium* cells in this region sensed the gradient and were chemotactically directed toward the entry and into the side branch. On their way, they picked up one or several 4.5 µm beads and moved their cargo into the narrow side channel toward the source of cAMP, see Figure 2E and Movies S7 and S8 in the Supporting Information. Thus, the cells readily completed a complex transport task that otherwise would require laborious operations such as, for example, the one by one movement of individual particles by optical tweezers.

Transport by single cells is limited in terms of cargo size due to the limited pulling force of an individual cell. Thus, transport of larger objects can be only achieved by the collective action of many cells. During the aggregation phase of the developmental cycle of *D. discoideum*, multicellular clusters and coherent streams of chemotactic *Dictyostelium* cells form spontaneously and move collectively toward the aggregation center. These directionally moving multicellular assemblies should in principle be able to transport also larger objects towards the aggregation center. To test the transport capacities of cell streams and clusters, we have established a microfabrication protocol to produce micro‐objects of different sizes and geometries, based on photolithography in combination with a subsequent lift‐off process. These objects are made of SU‐8 2025 photo resist, they are 25 µm thick, and have lateral dimensions ranging between 100 and 250 µm, see **Figure** **3** for examples.

**Figure 3:**
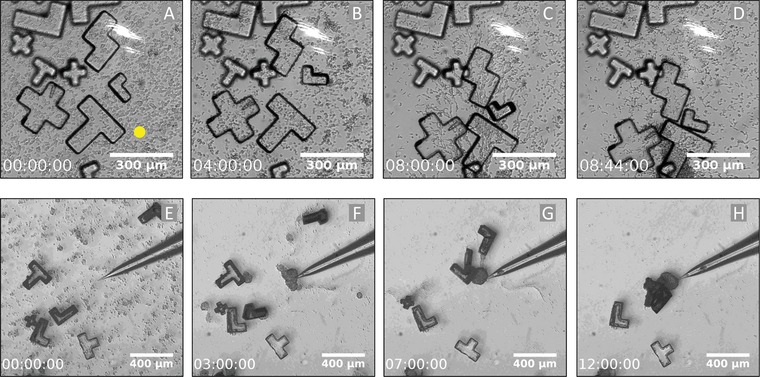
Chemotactically guided transport and assembly of micro‐objects by collectively moving cells and cell clusters. A–D) Cells are streaming towards a cAMP point source (position marked in panel (A)) and aggregate micro‐objects of different size and shape. The cAMP source was created by photochemical release from a BCMCM‐caged precursor with a 366 nm laser light spot. E–H) Chemotactic aggregation of cells and cell clusters at the tip of a cAMP releasing micropipette result in the assembly of micro‐objects of different size and shape.

We performed two types of experiments to investigate the collective transport of larger micro‐objects. First, starvation developed *Dictyostelium* cells were incubated together with a selection of micro‐objects of different shapes in BCMCM‐caged cAMP containing phosphate buffer in a liquid droplet on a microscope cover slip, see Figure 3A–D. To avoid liquid evaporation, the droplet was covered with a polydimethylsiloxane (PDMS) block that was fabricated with a 100 µm deep cavity on its surface (see the Supporting Information for details of this setup). Then, photouncaging was initiated with a 366 nm laser spot positioned in the bottom right hand part of the field of view. During the first eight hours, small clusters formed that eventually merged into streams moving toward an aggregation center in the vicinity of the uncaging spot. Our recordings show that the moving cell aggregates are indeed able to collectively transport micro‐objects that are more than one order of magnitude larger than the individual cells. Moreover, objects that are initially scattered over the surface are moved together and some of them even aligned side by side, see Figure 3A–D and Movie S9 in the Supporting Information.

In a second variant of this experiment, a glass micropipette filled with a solution of 100 µm of cAMP was used as a chemoattractant point source to guide the chemotactic motion, see Figure 3E–H. In this case, the recording was performed in a Petri dish, covered by a plastic lid with a small opening that allowed for access of the micropipette. Our data exemplifies how cell clusters that form at the edges of the micro‐objects carry these objects as they are chemotactically guided toward the micropipette. They may flip objects from one side to the other and can even lift them to an upright position. Finally, a tight assembly of the objects is formed with a large cell cluster at its center, see Figure 3H and the corresponding Movie S10 in the Supporting Information.

In summary, we demonstrated that motile amoeboid cells can be successfully harnessed as trucks to transport microcargo. When coming into contact with cargo particles, they pick up and move the micro‐objects, relying on their unspecific adhesive properties. Moreover, the transport process can be steered to desired target locations using the chemotactic signaling machinery of the amoeba. Here, the use of photoactivatable caged compounds enables chemotactic guidance even in closed environments that are difficult to access otherwise. Also larger pieces of cargo with several hundreds of micrometers in lateral extension can be moved and chemotactically guided relying on the collective action of large cell assemblies and chemotactic cell clusters. A major challenge for future work in this field will be to establish techniques for the controlled release of cargo. At the current stage, cells may lose cargo particles by accident or, alternatively, a current distribution of cargo particles can be frozen by killing the cellular microtrucks or by inhibiting their motility. For future applications we envision more elegant ways of programmable cargo delivery, such as the cleavage of specific cell‐cargo bonds induced by drugs or light.

Previously, other biohybrid strategies to transport microparticles have been proposed. In particular, inspired by intracellular transport mechanisms, transport assays relying on the interaction of molecular motors with cytoskeletal filaments were explored. Already several decades ago, particles coated with molecular motors were found to move along cytoskeletal fibers, such as actin filaments and microtubules.^32^, ^33^ Later, this type of motor‐driven translational motion was exploited in dedicated microtransport assays, for a review see Refs. 34, 35. Several initial limitations could be overcome, for example, unidirectional transport could be achieved by successfully preparing uniformly oriented arrays of the polar microtubules.^36^ However, compared to the cellular microtransporters presented here, the motor‐driven transport assays always require predefined tracks of cytoskeletal filaments that severely limit the directional flexibility of their motion.

Among the cell‐driven transport systems, the amoeboid microtrucks are in many respects complementary to the common biohybrid transport systems based on bacterial swimmers. While bacteria propel their cargo by swimming through a 3D fluid volume, the amoeboid trucks rely on substrate adhesion and move across 2D surfaces. For this reason, amoeboid transport systems will be less prone to perturbations by advective flows in the environment, while bacterial transporters may take advantage of advective transport to reach desired target locations more rapidly. Also the range of cargo sizes is complementary. The size of the objects that can be transported by an individual amoeba clearly exceeds the cargo that a bacterial swimmer could handle. While single *Dictyostelium* cells can move objects which are up to five times bigger than their own size, several bacteria are required to propel a micrometer sized bead.^37^ Finally, we note that amoeboid locomotion is a common mode of motility that is naturally encountered in different mammalian cell types, such as leukocytes, which makes the amoeboid microtrucks a promising concept for future medical applications.

## Experimental Section

*Cell Culture: D. discoideum* AX2 wild‐type cells were cultivated in HL5 medium (Formedium, Norwich, England) at 22 °C on polystyrene Petri dishes (Sarstedt, Nümbrecht, Germany), or shaken in suspension at 150 rpm. Suspensions of 4 × 10^4^ cells mL^−1^ and 1 × 10^4^ beads mL^−1^ (single cell carrying a single bead), 6 × 10^6^ cells mL^−1^ and 2 × 10^5^ beads mL^−1^ (ibidi chamber), 1.5 × 10^6^ cells mL^−1^ and 2 × 10^5^ beads mL^−1^ (uncaging dish), 1.5 × 10^7^ cells mL^−1^ and 2 × 10^7^ beads mL^−1^ (uncaging in microchannel), 2 × 10^5^ cells mL^−1^ (all other experiments) were used. Before the experiments of single cells carrying a single particle where carried out, the HL5 medium was renewed and cells and beads were distributed equally across the wells of a 24 well plate. The experiments started after an attachment time of 30 min. Prior to the other experiments, the cells were washed and transferred into 25 mL shaking phosphate buffer solution (150 rpm), composed of 14.6 mm KH_2_PO_4_ and 2 mm Na_2_HPO_4_ (Merck KGaA, Darmstadt, Germany) with a pH‐value of 6.0. In this solution the cell were starved. After ≈3 h (experiments in the gradient chamber and uncaging experiments in the microchannel and in the dish), or after ≈6 h (micropipette experiment and spontaneous aggregation experiments), or after ≈8 h (aggregation of the larger micro‐objects) the cells were centrifuged at 1000 rpm for 5 min (micropipette experiments), or 3 min (all other experiments). Afterwards, cells were mixed with a suspension of either polystyrene beads (Polysciences Europe GmbH, Hirschberg, Germany), or WGA coated agar beads (Bioworld, Dublin, OH 43 017, USA), or SU‐8 based micro‐objects to prepare them for the different experiments. For the photo uncaging experiments 10 µm BCMCM caged cAMP (BIOLOG Life Science Institute, Bremen, Germany) was added to the suspension. The cells were settled for 30 min inside the flow chambers or on the petri dishes to attach before the experiment was conducted.

*Image Acquisition and Processing*: For image acquisition three different microscopes have been used. For Figure 1A,B a laser scanning confocal microscope (LSM 780, Zeiss, Oberkochen, Germany), for Figure 3E–H a Olympus BX51WI (Olympus Deutschland GmbH, Hamburg, Germany) and for all others a Olympus IX71 (Olympus Deutschland GmbH, Hamburg, Germany) were used. As cameras an ORCA‐Flash 4.0 (Hamamatsu Photonics, Hamamatsu, Japan) was used for the experiments in the Ibidi chamber and the spontaneous bead assembly, an Olympus XM 10 (Olympus Deutschland GmbH, Hamburg, Germany) for the photouncaging experiments and a Hamamatsu R^2^ for the experiments with the micro objects. The time interval between frames in the experiments varies between ten and thirty seconds. To analyze the particle positions in Figure 1, we developed a Matlab (MathWorks, Natick, MA, USA) program based on the algorithm by Crocker and Grier (Crocker, Grier, 1996). To display the trajectory of the particle in Figure 2A ImageJ (NIH, Bethesda, MD, USA) was used.

*Gradient Generation*: cAMP gradient was generated in three different ways. To create a static linear gradient, an ibidi “µ‐Slide Chemotaxis” chamber (ibidi GmbH, Martinsried, Germany) was used. The gradient formed by diffusion between a reservoir filled with 10 µm cAMP and a reservoir filled just with buffer over a distance of 1 mm. To generate a chemical point source a micropipette filled with a phosphate buffer solution containing 100 µm cAMP was used. Furthermore a 366 nm UV‐Laser (Coherent, Santa Clara, CA, USA) was used to photochemically cleave the bond between the BCMCM caging group and cAMP and create a cAMP point source at the laser spot. A solution of 10 µm BCMCM caged cAMP in phosphate buffer was used for the uncaging experiments.

*Microfluidics*: The microfluidic channel was manufactured from PDMS by soft lithography as introduced by Duffy et al.^38^ For a detailed description of the process the interested reader is referred.^39^ Briefly, a silicon wafer was coated with SU‐8 2050 photo resist (micro resist technology GmbH, Berlin, Germany) in a vacuum spin coater (WS‐400BX‐6NPP/Lite, Laurell, North Wales, USA) to predefined height. After prebaking, the coated wafer was illuminated with a UV lamp (Tamarack PRX 500, San Francisco, USA) through a photomask (JD‐Phototools, Hitchin, United Kingdom). After postbaking, the structure was developed in dev‐600mr developer (micro resist technology GmbH, Berlin, Germany) until all nonilluminated photoresist was removed. After cleaning with 2‐propanol and drying at room temperature, the wafer was ready to use for the microchannel manufacturing process. For microfluidic chip fabrication, PDMS elastomer (Sylgard 184‐Kit, Dow Corning, Wiesbaden, Germany) was mixed with curing agent in a 10:1 ratio. The mixture of elastomer and curing agent was poured onto the structured silicon wafer and the PDMS was polymerized at 75 °C in a laboratory oven (Thermofischer, Germany). ≈5 h before the experiments started, the chip was cut with a scalpel out of the PDMS block on top of the wafer, inlet and outlet holes were punched into the PDMS with a syringe needle, and the PDMS block with the channel structure was plasma bonded to a glass cover slip in a vacuum plasma oven (Harrick, Ithaca, USA). Buffer solution was filled into a microsyringe (Harvard Apparatus, Holliston, USA), which was connected to the inlet in the PDMS microfluidic chip via PTFE tubings. The syringe was mounted onto a PHD Ultra micropump (Harvard Apparatus, Holliston, USA) and buffer solution was pumped through the microfluidic channel to get rid of air bubbles inside the system. Directly before the experiment started, the pump was switched off and the mixture of cells, beads, and caged cAMP was injected into the channel. During the experiment, no external buffer flow was added, to avoid that the beads are flushed away.

The chamber for photouncaging of cAMP to guide the aggregation of larger micro‐objects was made out of an oversize microscope glass cover slip (7.5 cm × 7.5 cm) and a PDMS block with a 2 cm × 1.7 cm × 100 µm cavity. A suspension of cells and micro‐objects was given on to the glass plate. Around this liquid droplet, a circle of a suspension of microbeads (75 µm in diameter) was placed with a diameter of 1 cm. The beads acted as spacers to avoid collapsing of the PDMS chamber. Finally, the PDMS block (≈3 cm × 4 cm × 0.5 cm) was placed on top and fixed on the glass with a halogen free oil (Baysilone Paste mittelviskos, Kurt Obermeier GmbH & Co.KG, Bad Berleburg‐Raumland, Germany), see the Supporting Information for a sketch of the setup.

*Fabrication of Microobjects*: To create the micro‐objects a similar protocol as for the PDMS master wafer was used. To remove the objects from the wafer, a teflon spatula was used. Before removing, the wafer was wetted with phosphate buffer. The removal should be done immediately after the development process, since bonding strength between wafer and photo resist increases with time. The objects can be stored in phosphate buffer or distilled water.

## Conflict of Interest

The authors declare no conflict of interest.

## Supporting information

Supplementary

Supplementary

Supplementary

Supplementary

Supplementary

Supplementary

Supplementary

Supplementary

Supplementary

Supplementary

Supplementary
