# Supplementary material for: Harnessing Motile Amoeboid Cells as Trucks for Microtransport and ‐Assembly
Source: Adv Sci (Weinh). 2018 Nov 28;6(3):1801242. doi: 10.1002/advs.201801242 (PMC6364505; doi:10.1002/advs.201801242)
Supplement: Supplementary file 1 — Supplementary [file ADVS-6-1801242-s002.pdf]

Copyright WILEY-VCH Verlag GmbH & Co. KGaA, 69469 Weinheim, Germany, 2018.

ADVANCED SCIENCE
Open Access

Supporting Information

for *Adv. Sci.,* DOI: 10.1002/advs.201801242

Harnessing Motile Amoeboid Cells as Trucks for Microtransport and -Assembly

*Oliver Nagel, Manuel Frey, Matthias Gerhardt, and Carsten Beta**

Copyright WILEY-VCH Verlag GmbH & Co. KGaA, 69469 Weinheim, Germany, 2016.

Supporting Information

Harnessing motile amoeboid cells as trucks for microtransport and -assembly

Oliver Nagel, Manuel Frey, Matthias Gerhardt, and Carsten Beta*

Supporting Figures

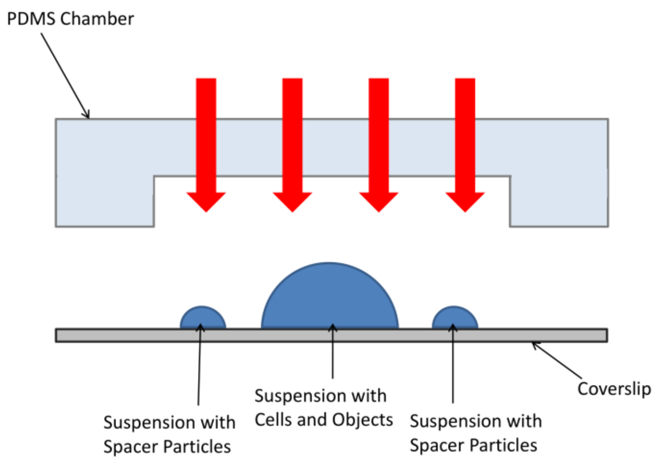

Figure S1: Schematic sketch of the assembly process of the microchamber used in Fig. 3A-D and Movie S9.

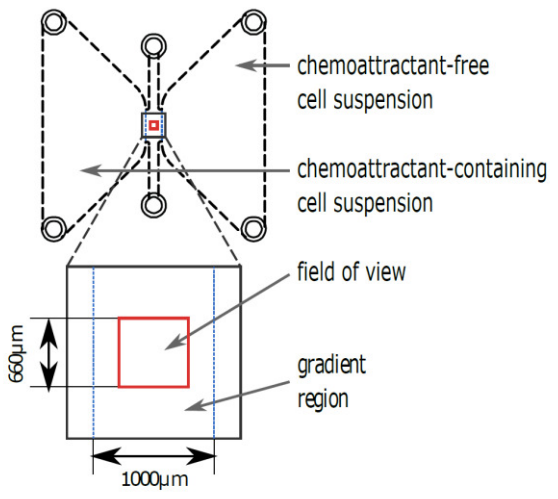

Figure S2: Schematic sketch of the ibidi "µ-Slide Chemotaxis" chamber used in Fig. 2A and Movie S5.

Supporting Movies

Movie S1: Cells moving randomly on a glass plate. When cells collide with a microbead, the bead stays attached to the cell and is carried along. Experiments have been conducted in HL5 medium in a Petri dish. For image acquisition we used an Olympus IX71 microscope with an ORCA-Flash 4.0 camera and 20x magnification. We took one image every 20 seconds, time is displayed in hours and minutes (hh:mm).

Movie S2: Microbeads of 10 µm in diameter in a Petri dish. Experiments have been conducted in phosphate buffer. For image acquisition we used an Olympus IX71 microscope with an ORCA-Flash 4.0 camera and 20x magnification. We took one image every 30 seconds, time is displayed in hours and minutes (hh:mm).

Movie S3: A randomly moving cell encounters a microbead and carries it along. The trajectory of the cell's center of mass is shown in red (displayed only until the collision with the bead) the trajectory of the bead is shown in turquoise. Experiments have been conducted in HL5 medium in a 24 well plate. For image acquisition we used a Zeiss LSM 780 with 20x magnification. We took one image every 30 seconds, time is displayed in hours and minutes (hh:mm).

Movie S4: Cells aggregating on a glass plate. While aggregating, the cells pick up microbeads resulting in an aggregation of the particles. Experiments have been conducted in phosphate buffer in a Petri dish. For image acquisition we used an Olympus IX71 microscope with an ORCA-Flash 4.0 camera and 4x magnification. We took one image every 30 seconds, time is displayed in hours and minutes (hh:mm).

Movie S5: Cells moving in a cAMP gradient inside an ibidi "µ-Slide Chemotaxis" chamber. The cAMP concentration increases from 0 (right) to 10µM (left) over a distance of 1 mm, see also Figure S2. On their way, the cells pick up microparticles and carry them along. For image acquisition we used an Olympus IX71 microscope with an ORCA-Flash 4.0 camera and 10x magnification. We took one image every 30 seconds, time is displayed in hours and minutes (hh:mm).

Movie S6: Cells moving on a glass plate towards a laser spot where cAMP is released by photo-uncaging. Experiments have been conducted in phosphate buffer containing 10µM BCMCM-cAMP in a glass bottom Petri dish. For image acquisition we used an Olympus IX71 microscope with an Olympus XM 10 camera and 20x magnification. We took one image every 30 seconds, time is displayed in hours and minutes (hh:mm).

Movie S7: Cells transporting microbeads into a narrow side channel towards a laser spot where cAMP is photo-chemically released. Experiments have been conducted in phosphate buffer containing 10µM BCMCM-cAMP. For image acquisition we used an Olympus IX71 microscope with an Olympus XM 10 camera and 20x magnification. We took one image every 30 seconds, time is displayed in hours and minutes (hh:mm).

Movie S8: Cells and cell aggregates transporting microbeads into a narrow side channel towards a laser spot where cAMP is photo-chemically released. Experiments have been conducted in phosphate buffer containing 10µM BCMCM-cAMP. For image acquisition we used an Olympus IX71 microscope with an Olympus XM 10 camera and 20x magnification. We took one image every 30 seconds, time is displayed in hours and minutes (hh:mm).

Movie S9: Cells streaming and aggregating on a glass plate towards a laser spot where cAMP is photo-chemically released. While aggregating, the cells transport and assemble microobjects of different size and shape. Experiments have been conducted in phosphate buffer containing 10µM BCMCM-cAMP inside a custom-made microchamber, see Figure S1. For image acquisition we used an Olympus IX71 microscope with a Hamamatsu R² camera and 10x magnification. We took one image every 30 seconds, time is displayed in hours and minutes (hh:mm).

Movie S10: Cells and cell clusters migrating towards a micropipette filled with a solution of 100µM cAMP. While aggregating at the pipette tip, they transport and assemble microobjects of different size and shape. Experiments have been conducted in phosphate buffer in a Petri dish. For image acquisition we used an Olympus BX51WI microscope with a Hamamatsu R² camera and 4x magnification. We took one image every 30 seconds, time is displayed in hours and minutes (hh:mm).
